# Fractional Carbon Dioxide Laser for Treatment of Microstomia and Rhytids in Systemic Sclerosis Patients

**DOI:** 10.31138/mjr.101223.fcd

**Published:** 2024-05-21

**Authors:** Elias Salimi, Shirin Assar, Aram Salimi, Dena Mohamadzadeh

**Affiliations:** Clinical Research Development Centre, Imam Reza Hospital, Kermanshah University of Medical Sciences, Kermanshah, Iran

**Keywords:** fractional CO2 laser, systemic sclerosis, microstomia, perioral rhytids

## Abstract

**Background:**

Systemic sclerosis (SSc) is an autoimmune disorder characterised by skin fibrosis leading to skin tightening and disfigurement. However, there is no definite treatment for SSc and its skin complications. Fractional carbon dioxide laser has been widely used for different cutaneous pathologies. This study aims to evaluate the benefits of CO2 laser resurfacing on microstomia and peri-oral rhytids in systemic sclerosis patients.

**Method and patients:**

33 systemic sclerosis patients were enrolled. Four sessions of CO2 laser treatment were performed at an interval of four weeks. Patients were evaluated monthly. The interincisal distance (IID) measurement was used to evaluate maximal mouth opening capacity, and the mouth handicap in systemic sclerosis (MHISS) scale was used to assess the improvement after treatment.

**Results:**

All of the participants were female with a mean age of 47.46 and a standard deviation of 7.11. The mean disease duration was 12.35. The mean total core of the MHISS scale was 25.24, and the mean IID was 48.11 millimetres before the treatment with CO2 laser. The MHISS score decreased, and patient satisfaction increased after treatment. However, observed differences in the results of IID were not statistically significant.

**Conclusion:**

In conclusion, it seems that the fractional carbon dioxide laser is effective in the improvement of perioral rhytids, patient satisfaction based on the MHISS score, and mouth disability in SSc patients.

## INTRODUCTION

Scleroderma refers to an autoimmune connective tissue fibrosing disease. Three different subsets of scleroderma have been introduced including localised scleroderma, limited cutaneous systemic sclerosis, and diffuse cutaneous systemic sclerosis. Systemic sclerosis (SSc) is characterised by vasculopathy and fibrosis of the skin and internal organs. Multiple organs including lungs, heart, kidneys, gastrointestinal tract, and blood vessels could be affected by SSc. Complications of SSc could be potentially fatal (such as renal crisis, and interstitial lung disease) or diminish the life quality of the patients.^1^ The earliest and most frequent manifestation of SSc is skin involvement which has three phases: oedematous, indurative, and atrophic phase. Skin manifestations include Raynaud’s phenomenon, skin sclerosis, nail fold changes, telangiectasia, hyperpigmentation, skin ulcerations, and calcification. The face is involved at an early stage of skin and soft tissue sclerosis. Facial skin changes are as follows: facial skin tightening, narrowing of the lips, microstomia, sharpening of the nose, and appearance of deep wrinkles around the lips (Rhytids). The mentioned facial changes lead to a loss of expressivity and a mask-like appearance. Though facial changes are not life-threatening, the disfigurement influences the patient’s quality of life.^2,3^ Although there is no definitive treatment, the first therapeutic options for progressive and diffuse cutaneous fibrosis are immune-modulating therapies such as methotrexate or mycophenolate mofetil. Cyclophosphamide is administered in refractory cases. Systemic steroids are not beneficial. Phototherapy has shown good results in the early phase of the limited and localised disease.^4,5^ Fractional carbon dioxide laser is a relatively new modality and has shown promising results in the treatment of morphea or localised scleroderma.^6^ The aim of this study was to investigate the therapeutic effect of carbon dioxide laser on scleroderma rhytids and microstomia.

## PATIENTS AND METHOD

This study was conducted at the Haj Daie Dermatology clinic and the Boostan Rheumatology clinic (Kermanshah University of Medical Sciences-associated clinics) in Kermanshah, Iran between June 22 and December 22, 2022. Convenience sampling was used to choose 33 patients from systemic sclerosis patients attending our rheumatology clinic for routine visits. Inclusion criteria were as follows: 1) patients met the American College of Rheumatology (ACR)/European League Against Rheumatism (EULAR) 2013 classification criteria for diffuse systemic sclerosis,^7^ 2) Age between 30–50, and exclusion criteria were as follows: 1) Allergy to the lidocaine or prilocaine cream, 2) patients had a tendency of keloid formation, 3) Patients were unable to complete four sessions of CO2 laser treatment for any reason. The patient’s medications did not change from six months before and during the study. The patients were advised not to undergo physical therapy or stretching exercises to improve mouth opening during the study and follow-up period. Demographic and clinical data including age, sex, and disease duration were recorded. Four sessions of CO2 laser treatment were performed at an interval of four weeks. Patients were evaluated monthly by expert rheumatologists and dermatologists for improvement and possible adverse events. The final evaluation was done after six months. The interincisal distance (IID) measurement was used to evaluate maximal mouth opening capacity (as described by Wood and Branco),^8^ before and after treatment by CO2 laser. The mouth handicap in systemic sclerosis (MHISS) scale was used to assess the improvement after treatment. MHISS is a 12-item questionnaire that measures mouth disability in SSc patients and has three subscales. Each item’s score ranges from 0 to 4 (zero for never, one for hardly ever, two for sometimes, three for usually, and four for always) and the total score ranges from 0 to 48. The subscales are reduced mouth opening, sicca syndrome, and aesthetic concerns. Higher scores indicate more disability. The results of IID and MHISS scales before and after treatment were compared. The CO2 laser procedure was as follows: If the patient had a previous history of herpes simplex, prophylaxis of infection associated with CO2 laser was performed by oral administration of valacyclovir (Virabex^®^) 1000 mg daily before the procedure and continuing until 3 days later. 70% alcohol solution was used to disinfect the treatment sites and then they were anesthetised with a topical cream containing 2.5% lidocaine and 2.5% prilocaine (Najo-caine P)^®^ under closed dressing, 30 minutes before the procedure.

Fractional CO2 laser [Edge one jeisys CO2 laser manufactured by South Korea], with 30 watts of power and a wavelength of 10,600 nm) treatment was performed in all patients in the perioral area. A gauze was placed between teeth and lips to prevent accidental toothburn. 120 sum handpiece was used. The spot density taken was 301(289 dots). The used fluence was 6.9 mJ/cm^2^. and pulse energy was 25Mj. The patients were given two passes without cleaning the epidermis between these two passes. At the end of the treatment, Zinc oxide 25% ointment was applied three times daily without dressing until complete healing.

Possible side effects of laser administration including pain, erythema, infection, hypo, or hyperpigmentation were recorded. The Clinical Research Ethics Committee of the Kermanshah University of Medical Sciences approved the study protocol (approval code: IR.KUMS.MED.REC.1401.079). All methods were carried out in accordance with the Declaration of Helsinki. Patients provided their informed written consent to participate in this study and for the publication of the results.

## STATISTICAL ANALYSIS

Collected data was inserted into SPSS software version 25. Quantitative variables (age, disease duration, and total score of the IID) were reported by mean and standard deviation, and we used paired T-test to compare quantitative variables (IID results) before and after treatment. Qualitative variables were reported as numbers and percentages and compared by Chi-square test. A P-value less than 0.05 was considered to be statistically significant.

## RESULTS

Thirty-three patients with systemic sclerosis were enrolled in the study. All of the participants were female with a mean age of 47.46 and a standard deviation of 7.11. The mean disease duration was 12.35 (the shortest three years, and the longest 20 years). The mean total core of the MHISS scale was 25.24, and the mean IID was 48.11 millimetres before the treatment with CO2 laser. **Table 1** contains demographical and clinical information of the patients.

**Table 1.** Demographical and clinical variables.

**Variables**	**Number (Percent)**	**Mean, Standard deviation**
**Gender**	33 (100)	
**Female**		
**Age**		47.46, 7.11
**Disease duration**		12.35, 4.51
**MHISS before CO2 laser treatment**		25.24, 8.26
**IID before CO2 laser treatment**		48.11, 6.28

Qualitative variables are reported by number and percent, and quantitative variables are reported by mean and standard deviation.

**Table 2** shows and compare the results of MHISS, and IID before treatment and after one month, two months, and six months after the beginning of treatment. The observed differences between the results of the MHISS scale are statistically significant (P-value= 0.01) which means that the mouth disability reported by the patients decreased after CO2 laser treatment. However, observed differences in the results of IID are not statistically significant (P-value= 0.58).

**Table 2.** The results of MHISS and IID scales compared before and after treatment.

	**Before treatment**	**One month after the first session**	**One month after the second session**	**Six months after the first session**	**P-value**
**MHISS result Mean SD**	25.24	20.65	18.53	18.29	0.01
**IID result (mm) Mean SD**	8.26	7.16	6.42	5.34	0.58

Qualitative variables are reported by number and percent, and quantitative variables are reported by mean and standard deviation.

**Table 3** compares the patient’s satisfaction and improvement status (based on the MHISS results). Patient satisfaction increased during the study (P-value= 0.001), and a greater proportion of the patients reported improvement after six months (P-value= 0.03).

**Table 3.** Patient satisfaction and improvement status one month, two months, and six months after the initiation of treatment.

	**One month after the first session**	**One month after the second session**	**Six months after the first session**	**P-value**
	*Number (percent)*	*Number (percent)*	*Number (percent)*	
**Patient’s satisfaction**				
**Poor**	17 (51.5)	10 (30.3)	6 (18.2)	0.001
**Moderate**	16 (48.5)	18 (54.5)	20 (60.6)	
**Excellent**	0	5 (15.2)	7 (21.2)	
**Improvement status**				
**Improved**	4 (12.1)	13 (39.4)	20 (60.6)	0.03
**Without change**	29 (87.9)	19 (57.6)	13 (39.4)	
**Worsened**	0	1 (3)	0	

**Figure 1 and Figure 2.**
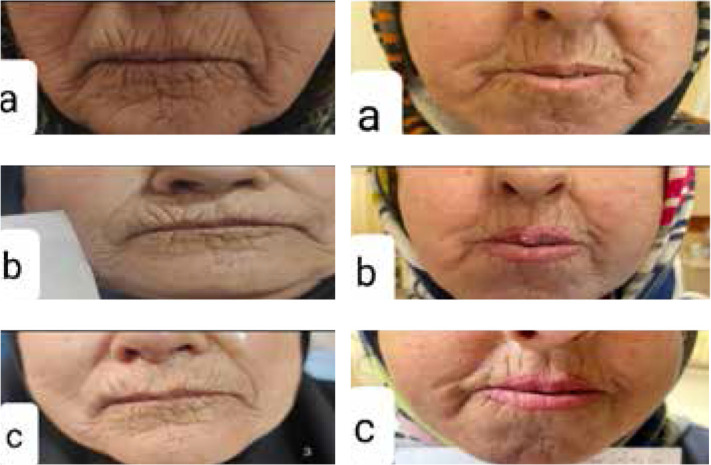
Showing the perioral rhytids of a 50 -year-old and a 44-year-old female with systemic sclerosis, respectively. **(a)** Before CO2 laser treatment. **(b)** One month after the first session of laser treatment. **(c)** After six months.

**Figure 1** and **Figure 2** show the two patients’ perioral rhytids before and after the treatment with CO2 laser.

Possible side effects were investigated after each laser session. Mild pain and self-limiting erythema were the only side effects. No serious complication was reported.

## DISCUSSION

In this survey, we included 33 SSc patients. They were all treated by carbon dioxide laser for perioral rhytids in four sessions at four-week intervals. They were evaluated by MHISS and IID monthly and after six months. Significant improvement was reported subjectively by the patients based on MHISS, but although the interincisal distance increased objectively during the study, it was not statistically significant and none of the patients had complications during the survey.

Fibrosis of the skin which occurs in almost all patients with diffuse systemic sclerosis leads to many cosmetic and functional problems. Skin fibrosis leads to radial furrowing around the lips and thinning of them and therefore, it leads to the inability to fully open the mouth and difficulty in oral and dental hygiene. Unfortunately, until now there is no effective treatment to prevent or eliminate these skin changes. Today’s fractional CO2 laser is used for the treatment of many skin diseases related to tissue fibrosis. This technology has allowed us to effectively correct aesthetic defects, such as scars, hyperpigmentation, and superficial or deep rhytids.^9.10^ It also provides a new treatment for scleroderma and other connective tissue diseases, that often cause permanent aesthetic defects.^11^

The explained mechanisms of CO2 laser on collagen remodelling are as follows: a. removal of homogenised and fibrotic tissue, releasing the skin tightness, b. the long-term effect on dermal collagen remodelling, c. degradation of thickened collagen bundles, c. modulating the expression of transforming growth factor beta 1 (TGFb1, a pro-fibrotic mediator) and vascular endothelial growth factor (a neo-angiogenesis mediator).^12–20^ Fractional ablative laser is based on fractional photothermolysis. This leads to an immediate mechanical effect that removes fibrotic tissue and releases skin tightness.^12^ Controlled heat exposure is followed by a wound-healing response that ultimately leads to long-term skin remodelling, thereby reducing TGFb levels and causing induction of the expression of metalloproteinases (MMP-1, MMP-3, MMP-9, and MMP-13) that are responsible for the degradation of inappropriate homogenised collagen fibers.^13–17^ TGFb1 is a well-known fibrogenic mediator that plays an important role in the pathogenesis of scleroderma by disrupting the balance between collagen formation and degradation, thereby depositing extracellular matrices.^18–20^ There is also an increase in heat shock proteins (HSPs) in response to injury, particularly in the early stages of healing after laser treatment, with an increase in HSP-72 and an increase in HSP-47 up to 3 months after the session. HSP-72 leads to the stimulation of epidermal stem cells and initiation of dermal collagen remodelling, and HSP-47 affects fibroblasts and enables long-term collagen remodelling. slowly and irregularly, the arrangement of collagen bundles is replaced by a new arrangement of ordered fibers. About 1 month after treatment, type 3 collagen is replaced with type 1.^13–20^

Apfelberg et al. reported three patients with SSc who were treated by a carbon dioxide laser. This was the first article that investigated the effect of CO2 laser on perioral wrinkles in SSc patients. They reported significant improvement and no obvious complications.^21^ Bennani et al reported four SSc patients with systemic sclerosis with severely limited mouth opening (IID< 30mm) treated by one to three CO2 laser sessions. A mean gain of +5 mm in IID was observed three months after the first session and a mean gain of +8.5 mm after 12 months.^22^ In our study, the mean IID was 48.^11^ before the treatment which reached 50.17 one month after the first session, and 50.70 at the end of the study (after six months). The increase in IID was not statistically significant in our study. We found a mean decrease of −7 in the MHISS score which was statistically significant and 60.65% of the patients reported improvement which is consistent with the study of Bennani et al. reported a mean decrease of −14 in the MHISS score.

The fractional CO2 laser was also used for localised scleroderma (morphea). Szczepanik-Kulak et al. performed a systematic review of the literature and recognised a total of twenty previous studies that used laser therapy for localised scleroderma, among them six studies were related to fractional lasers (CO2 and Er: YAG). The mentioned six studies included a total of 24 cases of morphea treated with CO2 laser. The first case was a 27-year-old female with linear morphea which caused flexion contracture of her foot. One session of treatment with a CO2 laser resulted in a remarkable recovery. This review article concluded that laser therapy neither slows down the overall course of morphea nor prevents the formation of new lesions.^23^ In different studies, according to the type and method of laser and the number of sessions, different results are expected. Our study has both strengths and limitations. The main strength of this study is the large sample size compared to the previous studies which allowed us to drive a more accurate conclusion. Also, we used the MHISS scale which is a validated scale to evaluate the improvement of mouth disability in SSc patients. The main limitation is that the MHISS scale is a subjective and Self-administrating scale. Further studies with a larger sample size using additional objective methods to assess clinical response to CO2 laser treatment in SSc patients are required.

## CONCLUSION

It seems that the fractional carbon dioxide laser is effective in the improvement of perioral rhytids, and the patient’s satisfaction based on the MHISS score, although further studies are needed to determine the best strategy for the fractional CO2 laser.

## Data Availability

Data is available if requested.
